# Pharmacologically active flavonoids from the anticancer, antioxidant and antimicrobial extracts of *Cassia angustifolia* Vahl

**DOI:** 10.1186/s12906-016-1443-z

**Published:** 2016-11-11

**Authors:** Shabina Ishtiaq Ahmed, Muhammad Qasim Hayat, Muhammad Tahir, Qaisar Mansoor, Muhammad Ismail, Kristen Keck, Robert B. Bates

**Affiliations:** 1 Department of Plant Biotechnology, Atta-Ur-Rahman School of Applied Biosciences (ASAB), National University of Sciences and Technology (NUST), H-12 Islamabad, Pakistan; 2 Institute of Biotechnology and Genetic Engineering (IBGE), Abdul Qadeer Khan Research Laboratory (KRL) Hospital, Islamabad, Pakistan; 3 Bio5, University of Arizona, Tucson, AZ USA; 4 Department of Chemistry and Biochemistry, University of Arizona, Tucson, AZ USA

**Keywords:** *Cassia angustifolia*, Antimicrobial, Antioxidant, Anticancer, Flavonoids

## Abstract

**Background:**

*Cassia angustifolia* Vahl. (commonly known as senna makkai or cassia senna), native to Saudi Arabia, Egypt, Yemen and also extensively cultivated in Pakistan, is a medicinal herb used traditionally to cure number of diseases like liver diseases, constipation, typhoid, cholera etc. This study was conducted to evaluate the in-vitro antimicrobial, antioxidant and anticancer assays and phytochemical constituents of aqueous and organic extracts of *C. angustifolia* leaves.

**Methods:**

The antimicrobial activities of *C. angustifolia* aqueous and organic (methanol, ethanol, acetone, ethyl acetate) extracts were investigated by the disk diffusion method. These extracts were further evaluated for antioxidant potential by the DPPH radical scavenging assay. Anticancer activities of the extracts were determined by the MTT colorimetric assay. The total phenolic and flavonoid contents of *C. angustifolia* extracts were evaluated by the Folin-Ciocalteu method and aluminum chloride colorimetric assay, respectively. The structures of the bioactive compounds were elucidated by NMR and ESI-MS spectrometry.

**Results:**

Bioactivity-guided screening of *C. angustifolia* extracts, led to the isolation and identification of three flavonoids quercimeritrin (**1**), scutellarein (**2**), and rutin (**3**) reported for the first time from this plant, showed significant anticancer activity against MCF-7 (IC_50,_ 4.0 μg/μL), HeLa (IC_50,_ 5.45 μg/μL), Hep2 (IC_50,_ 7.28 μg/μL) and low cytotoxicity against HCEC (IC_50,_ 21.09 μg/μL). Significant antioxidant activity was observed with IC_50_ 2.41 μg/mL against DPPH radical. Moreover, *C. angustifolia* extracts have the potential to inhibit microbial growth of *E. cloacae*, *P. aeruginosa*, *S. mercescens* and *S. typhi*.

**Conclusion:**

*C. angustifolia* extracts revealed the presence of quercimeritrin (**1**), scutellarein (**2**), and rutin (**3**), all known to have useful bioactivities including antimicrobial, antioxidant and anticancer activities.

## Background

In recent years, there has been an alarming increase in the antibiotic resistance to a broad range of human pathogenic bacterial and fungal strains which contribute to the recurrence of infectious diseases. Urinary tract, bloodstream, and respiratory organs are some of the common sites of infections caused by antibiotic resistance microbes throughout the world [1]. Multidrug-resistant bacterial and fungal strains are the main cause of hospital acquired infections which reduce the efficacy of drugs and are ultimately responsible for treatment failure [2]. This situation has created a need to find more effective drugs. Natural products from microorganisms have been the primary source of antibiotics, and with the increasing acceptance of herbal medicines, the screening of medicinal plants for new active compounds has become a very important source of novel antibiotics [3].

Extensive researches on medicinal plants have also indicated that they are good sources of antioxidants [4–6]. They are involved in free radical scavenging activities which contribute to the protection from oxidative stress caused by the overproduction of free radicals and reactive oxygen species [7]. These are produced as byproducts of various biochemical and physiological processes in the human body [8]. Biomolecular body systems such as lipids, DNA, RNA and proteins are adversely affected by the oxidative stress which eventually leads to human chronic diseases such as Alzheimer’s, cardiovascular, atherosclerosis, cancer, stroke, fibrosis, aging and diabetes [9]. According to many research reports, the consumption of medicinal plants either in the form of raw extracts or chemical constituents is largely associated with lower risk of degenerative diseases caused by oxidative stress because they contain antioxidants such as phenolics, flavonoids, vitamins and carotenoids [10]. Phenolic compounds such as phenolic acids and flavonoids are reported to be involved in various biochemical activities like antioxidant, antimicrobial, antithrombotic, antiartherogenic, anti-inflammatory, anticarcinogenic and antimutagenic [11]. Natural antioxidants of plant origin are usually more potent and beneficial than synthetic antioxidants such as propylgallate (PG), butylated hydroxytoluene (BHT), t-butylhydroxytoluene (TBH) and butylated hydroxyanisole (BHA) [12]. It was also reported that synthetic antioxidants were the cause of carcinogenesis and liver damage in laboratory animals [13]. Thus there is a need to explore and develop antioxidants of natural origin with greater efficacy and fewer side effects.

Cancer is the second largest cause of death worldwide [14]. Although great advancements have been made in the treatment and control of cancer progression, significant deficiencies and room for improvement remains. A number of undesired side effects sometimes occur during chemotherapy. Natural therapies, such as the use of plant-derived products in cancer treatment, may reduce adverse side effects. There are many natural products including phytochemicals and dietary compounds from vegetables, plants, spices and herbs that have been used for the treatment of cancer throughout history due to their safety, low toxicity, and general availability [15].

*C. angustifolia* Vahl. is a traditional medicinal plant belonging to the family Caesalpiniaceae. It is commonly known as senna makkai or cassia senna. *C. angustifolia* is native to Saudi Arabia, Egypt, and Yemen. It is a rapid-growing shrub 5–8 m tall, extensively cultivated for its fruit and leaves in hot arid areas of Pakistan [16]. This plant is recognized in British and USA pharmacopoeias [17]. The leaves and pods of *C. angustifolia* are used in the form of a decoction powder for intestinal worms as an anti-helmenthic. It is also widely used as an anti-pyretic in typhoid, splenic enlargements, cholera, laxative, anemia, toxicity and genotoxicity caused by *Escherichia coli* [16].

Worldwide consumption of *C. angustifolia* as a folk medicine against various ailments and the studies reported in the literature demand further research to discover the compounds responsible for its bioactivities. This research study was conducted to investigate the antibacterial, antifungal, antioxidant, and anticancer potentials of aqueous as well as organic extracts of *C. angustifolia*. They were also subjected to phytochemical screening to determine the presence of secondary metabolites and bioactive compounds.

## Methods

### Formulation of plant crude extracts

*C. angustifolia* seed powder were bought from a local herb shop in Islamabad and is identified by taxonomist Dr. Muhammad Qasim Hayat, ASAB (Atta-ur-Rahman School of Applied Biosciences), NUST (National University of Sciences and Technology), Islamabad. It was identified by comparing with the voucher specimen no P03088812 of Herbier museum Paris (http://mediaphoto.mnhn.fr/media/1441330963480e0yHgeqQ0CSk9mGW). The local botanical description of *C. angustifolia* is also available at flora of Pakistan (http://www.tropicos.org/Name/13028414?projectid=32) and original plant material was kept at MPRL (Medicinal Plant Research Laboratory), ASAB, NUST for future references. Extracts were formulated by maceration. The finely ground powder (mesh size 50 = 0.297 mm) was subjected to aqueous and organic solvents (methanol, ethanol, acetone, and ethyl acetate) separately in flasks with the ratio 1:10 and placed in the dark at 37 °C for three days with intermittent shaking. Centrifugation was performed for 15 min at 2000 rpm. Supernatants were filtered with Whatman filter paper no.1. The filtrate was transferred to a round-bottom flask and the solvent was rotary evaporated. The dry extract was stored at 4 °C.

### Bacterial and fungal strains

*Acinetobacter junii* IARS2, *Serratia mercescens* IARS6, *Enterobacter cloacae* IARS7, *Pseudomonas aeroginosa* IARS8 and *Salmonella typhi* ATCC 14079 were used in antibacterial assays. Glycerol stocks of all the bacterial strains were maintained in controlled conditions and subcultured on Mueller Hinton agar for 24 h before antibacterial assay. The fungal strain *Candida albicans* was obtained from the Institute of Biotechnology and Genetic Engineering (IBGE), Abdul Qadeer Khan Research Laboratory (KRL) Hospital, Islamabad. It was maintained at 37 °C on Sabouraud dextrose agar.

### Antimicrobial assay

Organic and aqueous extracts of *C. angustifolia* were screened for antibacterial and antifungal potential by disk diffusion assay as previously reported [18].

### Antioxidant assay

The antioxidant activities of the organic and aqueous extracts of *C. angustifolia* and gallic acid were evaluated with the DPPH method as previously described [19]. Antioxidant potential of *C. angustifolia* extracts were analyzed and mentioned as IC_50,_ which was calculated from the calibration curve of the standards by using MS Excel 2010.

## Anticancer assay

### Cell culture

The HeLa was provided by HBV (Hepatitis B Virus) lab, ASAB, NUST. Hep2, MCF-7, and HCEC were provided by IBGE, KRL Hospital, Islamabad. All the cell lines were grown in RPMI-1640 media which contained 10 % FBS. All cells were maintained at 37 °C in a humidified atmosphere of 5 % CO_2_.

### MTT assay

Anticancer assay of *C. angustifolia* on Hep2, HeLa, MCF-7, and HCEC were carried out by the MTT colorimetric assay as employed earlier [20]. The anticancer activities of each tested extract were presented as IC_50_, which was calculated by percentage cell death at 100 μg/μL, 150 μg/μL, 200 μg/μL, 250 μg/μL using MS excel 2010.

### Qualitative phytochemical screening for secondary metabolites

*C. angustifolia* extracts were phytochemically screened for the presence of secondary metabolites. Qualitative phytochemical analyses for the presence of steroids, alkaloids, tannins including phlobatannins, monoterpenes, flavonoids, coumarins, cardiac glycosides, saponins, diterpenes, anthraquinones, and phenols were carried out by standard protocols [21–23].

### Determination of total phenolic contents

Total phenolic contents of the organic and aqueous extracts of *C. angustifolia* were determined by the Folin-Ciocalteu method [24].

### Determination of total flavonoid contents

The total flavonoid contents of *C. angustifolia* extracts were determined by the aluminum chloride colorimetric method [19].

### Isolation of active compounds

The *C. angustifolia* extracts were submitted to HPLC-MS analysis for screening of active compounds. A Shimadzu preparative HPLC, equipped with an LC-20 AD pump (the make-up pump), two LC-8A pumps (the gradient pumps), an SPD-20A UV detector and a CTC analytics PAL sample injector and fraction collector were used for the isolation of compounds. The fractions were collected based on UV @ 214 nm. The column used was a Phenomenex Gemini-NX C18 (30 × 50 mm i.d., 5 μm particle size, 110 A). The injection volumes were 800 to 1200 μL. The gradient was 5 to 70 % acetonitrile over 25 min with a flow rate of 35 mL/min. The modifier was 0.1 % formic acid, used primarily for ionization.

### Identification of active compounds

The structural identifications of isolated compounds were carried out by ^1^H NMR analysis. ^1^H NMR data were obtained by using a Bruker AVANCE III 400 MHz. NMR spectrometer equipped with a PA BBO 400S1 probe and a sample jet autosampler.

### Statistical analysis

All the experiments were carried out in triplicate. Data were presented as mean ± SD. *T*-Test was performed to determine statistical significance. Microsoft Excel 2010 was used for the statistical and graphical evaluations.

## Results

### Antimicrobial activity

The antibacterial activities of different extracts of *C. angustifolia* and controls are shown in Tables 1 and 2. All the tested pathogenic bacterial strains were sensitive to *C. angustifolia* extracts. Extracts of *C. angustifolia* showed variable degrees of bactericidal activity, with inhibitory effects of methanol extracts being observed against all of the selected bacterial strains. The highest bactericidal activity of the ethyl acetate extract was recorded against *S. mercescens*, with a 10.5 ± 0.76 mm zone of inhibition at 1.25 mg/mL. It was observed that *A. junni*, *E. cloacae* and *P. aeroginosa* were resistant to aqueous extract. The ethanol extract showed no antibacterial activity against *E. faecalis* and *P. aeruginosa*, while it showed its best antibacterial activity against *S. mercescens* with a 9.0 ± 0.50 mm zone of inhibition at 1.25 mg/mL. *A. junii*, *S. mercescens*, *E. cloacae*, and *S. typhi* were sensitive to acetone extract. Tigecycline, amikacin and cefepime (5 μg/mL) were used as standard antibiotic drugs (Table 2).

**Table 1:** Zone of inhibition of *C. angustifolia* extracts against pathogenic bacterial and fungal strains

Microrganisms	Extracts	Zones of Inhibition (mm)			
		1.25 mg/mL	2.5 mg/mL	5 mg/mL	10 mg/mL
*Acinetobacter junii*	Methanol	0.0 ± 0.0	0.0 ± 0.0	7.5 ± 0.1**	10 ± 0.2**
	Acetone	0.0 ± 0.0	0.0 ± 0.0	7.9 ± 0.1**	9.5 ± 0.1**
	Ethyl acetate	0.0 ± 0.0	8.0 ± 0.2**	10.5 ± 0.2**	11 ± 0.36**
	Ethanol	0.0 ± 0.0	0.0 ± 0.0	8.0 ± 0.36**	10 ± 0.2**
	Aqueous	0.0 ± 0.0	0.0 ± 0.0	0.0 ± 0.0	0.0 ± 0.0
*Serratia mercescens*	Methanol	8.5 ± 0.2**	8.8 ± 0.3**	10.8 ± 0.26*	11.6 ± 0.2**
	Acetone	9.5 ± 0.36**	9.7 ± 0.17**	10.5 ± 0.60*	12.8 ± 1**
	Ethyl acetate	10.5 ± 0.26*	10.8 ± 0.1**	11.6 ± 1.2*	12.5 ± 1.1*
	Ethanol	9.0 ± 0.60**	10.0 ± 0.17**	12.1 ± .95*	13.3 ± 0.51**
	Aqueous	7.3 ± 0.33**	7.8 ± 0.57**	9.0 ± 0.60**	10.0 ± 0.20**
*Enterobacter cloacae*	Methanol	7.3 ± 0.26**	7.5 ± 0.20**	8.6 ± 0.36**	10.6 ± 0.20**
	Acetone	0.0 ± 0.0	0.0 ± 0.0	7.5 ± 0.3**	8.5 ± 0.1**
	Ethyl acetate	7.8 ± 0.78*	8.5 ± 0.1**	10.5 ± 0.26**	12.5 ± 0.50**
	Ethanol	0.0 ± 0.0	0.0 ± 0.0	0.0 ± 0.0	0.0 ± 0.0
	Aqueous	0.0 ± 0.0	0.0 ± 0.0	0.0 ± 0.0	0.0 ± 0.0
*Pseudomonas aeruginosa*	Methanol	7.5 ± 0.20**	8.5 ± 0.1**	10.0 ± 0.20**	12.1 ± 0.43**
	Acetone	0.0 ± 0.0	0.0 ± 0.0	0.0 ± 0.0	0.0 ± 0.0
	Ethyl acetate	0.0 ± 0.0	0.0 ± 0.0	0.0 ± 0.0	0.0 ± 0.0
	Ethanol	0.0 ± 0.0	0.0 ± 0.0	0.0 ± 0.0	0.0 ± 0.0
	Aqueous	0.0 ± 0.0	0.0 ± 0.0	0.0 ± 0.0	0.0 ± 0.0
*Salmonella typhi*	Methanol	7.8 ± 0.3**	8.8 ± 0.52**	10.8 ± 0.65**	12.17 ± 0.43**
	Acetone	7.1 ± 0.6*	7.8 ± 0.30**	8.2 ± 0.36**	8.9 ± 0.43**
	Ethyl acetate	8.8 ± 0.28**	9.3 ± 0.45**	10.0 ± 0.20**	12.5 ± 0.62**
	Ethanol	7.1 ± 0.26**	8.0 ± 0.34**	10.5 ± 0.50**	11.8 ± 0.40**
	Aqueous	7.6 ± 0.40**	8.8 ± 0.40**	9.5 ± 0.43**	10.0 ± 0.34**
*Candida albicans*	Methanol	8.5 ± 0.30**	10.5 ± 0.78**	11.5 ± 0.36**	12.0 ± 0.8**
	Acetone	10 ± 0.34**	0.0 ± 0.0	0.0 ± 0.0	0.0 ± 0.0
	Ethyl acetate	9.0 ± 0.20**	9.5 ± 0.51**	10 ± 0.20**	0.0 ± 0.0
	Ethanol	10.0 ± 0.1**	10.8 ± 0.40**	11.0 ± 1.3*	11.3 ± 0.55**
	Aqueous	0.0 ± 0.0	0.0 ± 0.0	0.0 ± 0.0	0.0 ± 0.0

Each value represents the mean ± standard deviation of three replicates (*n* = 3)

(*), and (**), significant at level *P* ≤ 0.01 and 0.001, respectively

**Table 2:** Zone of inhibition of positive and negative controls against pathogenic bacterial and fungal strains

Controls	Chemicals	Zone of Inhibition (mm)					
		*A. junii*	*S. mercescens*	*E. cloacae*	*P. aeroginosa*	*S. typhi*	*C. albicans*
Positive	Tigecycline Amikacin Cefepime	12.1 ± 0.2 8 ± 0.20 0.0 ± 0.0	15.0 ± 0.50 13.3 ± 0.28 0.0 ± 0.0	16.1 ± 0.28 20.1 ± 0.28 19.8 ± 0.28	12.5 ± 0.50 12.0 ± 0.50 0.0 ± 0.0	16.0 ± 0.50 20.1 ± 0.29 17.0 ± 0.50	13.2 ± 0.2 0.0 ± 0.0 12.1 ± 0.3
Negative	DMSO^(a)^	0.0 ± 0.0	0.0 ± 0.0	0.0 ± 0.0	0.0 ± 0.0	0.0 ± 0.0	0.0 ± 0.0

Values are expressed as mean ± SD (*n* = 3). (^a^) Dimethylsulfoxide

The antifungal potential of the extracts were measured in terms of clear zone of inhibition of fungal growth (Table 1). All the tested *C. angustifolia* extracts had significant antifungal activity. The methanol extract exhibited the highest antifungal activity with a zone of inhibition of 12 mm at 10 mg/mL as compared to the standard amikacin drug which is sensitive to fungal growth. The aqueous extract showed no significant antifungal activity while the ethanol and ethyl acetate extracts showed moderate antifungal activity with zones of inhibition of 11 and 10 mm, respectively.

### Antioxidant assay

**Fig. 1:**
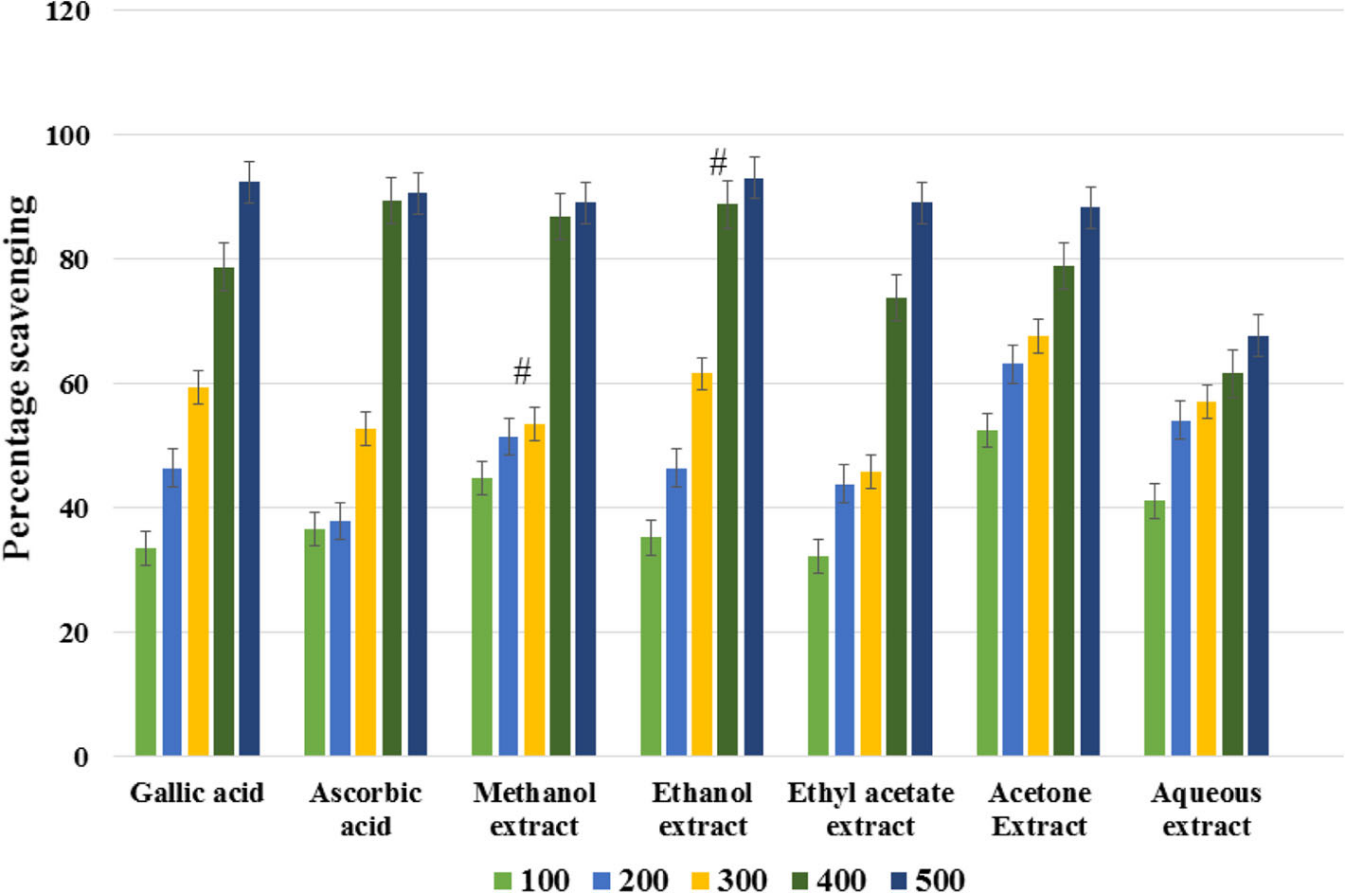
Graph showing DPPH radical scavenging activity of different extracts of *C. angustifolia*. Each point represents the mean of three experiments. Data are expressed as mean ± standard deviation. (#) indicated values are not significant at *P* < 0. 05

The antioxidant activities of the aqueous and organic extracts of *C. angustifolia* and gallic acid were evaluated by the free radical DPPH scavenging test on the basis of IC_50_ values (Table 3). IC_50_ values are the inhibitory concentrations required for 50 % scavenging of DPPH free radicals. The smaller the IC_50_ values, the higher the antioxidant potential of the plant constituents. The absorbance values of different extracts of *C. angustifolia* and standards were measured at the wavelength 517 nm. The resulting absorbance of the extracts give percentage scavenging of DPPH free radicals. All the extracts have dose dependent antioxidant activities, i.e., the scavenging activities of the extracts increased with the respective increase in the concentrations (Fig. 1). According to the results, all the extracts have potential antioxidant activities. The ethanol extracts exhibit maximum DPPH free radical scavenging activity (93 %) at a concentration of 500 μg/mL with an IC_50_ value of 2.41 ± 0.02 μg/mL, whereas the aqueous extract showed poor DPPH scavenging activity (68 %) at 500 μg/mL with IC_50_ values of 3.03 ± 0.04 μg/mL. The other *C. angustifolia* extracts showed moderate DPPH scavenging activities (Fig. 1).

**Table 3:** IC_50_ of free radical scavenging activity of standards and different extracts of *C. angustifolia*

Test material		DPPH radical IC_50_ in μg/mL
Plant extracts	Methanol	2.49 ± 0.01*
	Ethanol	2.41 ± 0.02*
	Acetone	3.06 ± 0.04**
	Ethyl acetate	2.74 ± 0.02*
	Aqueous	3.03 ± 0.04**
Standards	Gallic acid	2.52 ± 0.02
	Ascorbic acid	2.54 ± 0.00

Each value represents the mean ± standard deviation of three replicates (*n* = 3)

(*), and (**), significant at level *P* ≤ 0.01 and 0.001, respectively

### Anticancer assay

The cytotoxic studies for *C. angustifolia* extracts employed the MTT colorimetric method. The *C. angustifolia* aqueous, methanol, ethanol, acetone and ethyl acetate extracts were investigated first time for their anticancer potential against Hep2, HeLa, MCF-7 and normal HCEC cell lines. Only the ethanol extract exhibited anticancer activity, with 28 % death in Hep2 cells with an IC_50_ of 7.28 μg/μL. Methanol and ethanol extracts exhibited 33 and 23 % cell death in HeLa cell lines and 43 and 23 % cell death in MCF-7 cell lines (Table 4). The IC_50_ values are used to find the potency of drugs, lower IC_50_ values mean more potent the drugs. It was observed that IC_50_ value of methanol extract is 5.45 μg/μL against HeLa cells and 4 μg/μL against MCF-7 cells which are far less than the IC_50_ value of standard anticancer drug taxol 6.07 μg/μL and tamoxifen 6.4 μg/μL. *C. angustifolia* methanol and ethanol extracts were further examined for cytotoxicity against the normal cell line to answer whether it is selective towards cancerous cells. For this, normal HCEC cells were incubated with different concentrations of methanol and ethanol extracts (100, 150, 200 and 250 μg/μL) to analyze cell viability. The data showed that HCEC cells were unaffected by exposure to methanol and ethanol extracts. It showed ca. 100 % cell viability against different concentrations of methanol and ethanol extract. These results showed that methanol and ethanol extracts have the potential to inhibit the proliferation of Hep2, HeLa, and MCF-7 cells. These cells were more sensitive to inhibition by *C. angustifolia* extract than normal HCEC cell lines.

**Table 4:** Anticancer activity of *C. angustifolia* extracts against HCEC, Hep2, HeLa, and Mcf-7cell lines

Cell Lines	Extracts	% Cell death				I.C_50_
		100 μg/μL	150 μg/μL	200 μg/μL	250 μg/μL	μg/μL
HCEC	Methanol	2.3 ± 0.76	1.83 ± 0.76	3.43 ± 0.50*	13.7 ± 2.42*	21.09
	Ethanol	1.2 ± 0.52	6.23 ± 1.02*	9.20 ± 0.81*	12.8 ± 1.01*	16.23
	Taxol	0.0 ± 0.0	0.0 ± 0.0	0.0 ± 0.0	0.0 ± 0.0	0.0
	Tamoxifen	14.5 ± 0.5	19.3 ± 0.7	26 ± 0.5	26.8 ± 0.7	6.29
Hep2	Ethanol	6.06 ± 0.49**	11.83 ± 0.65**	21.0 ± 0.90*	28.26 ± 0.25*	7.28
	Taxol	26.5 ± 0.5	31.76 ± 0.66	35.53 ± 0.25	45.16 ± 0.60	3.97
HeLa	Methanol	16.7 ± 0.70**	22.56 ± 0.40**	27.73 ± 0.2**	32.46 ± 0.61**	5.45
	Ethanol	5.46 ± 0.40**	15.53 ± 0.37**	18.43 ± 0.4**	22.9 ± .075**	8.18
	Taxol	11.46 ± 0.40	19.7 ± 0.51	24.63 ± 0.50	30.56 ± 0.45	6.07
MCF-7	Methanol	23.33 ± 0.7**	26.23 ± 0.25**	42.4 ± 0.36**	43.6 ± 0.45**	4.0
	Ethanol	6.8 ± 0.52*	11.5 ± 0.37**	16.1 ± 0.1**	23.56 ± 0.45**	8.79
	Tamoxifen	12.46 ± 0.40	17.36 ± 0.55	24.63 ± 0.50	28.56 ± 0.45	6.4

Taxol and Tamoxifen are standard anticancer drugs taken as positive controls. Values represents the mean ± standard deviation of three replicates (*n* = 3)

(*), and (**), significant at level *P* ≤ 0.01 and 0.001, respectively

### Phytochemical screening

Qualitative phytochemical screening of different extracts of *C. angustifolia* revealed the presence of steroids, alkaloids, tannins including phlobatannins, monoterpenes, flavonoids, coumarins, cardiac glycosides, saponins, diterpenes, anthraquinones, and phenols which contribute to the antimicrobial, antioxidant and anticancer activities of the plant (Table 5).

**Table 5:** Phytochemical screening for secondary metabolites of different solvent extracts of *Cassia angustifolia*

EXTRACTS	Methanol	Ethanol	Acetone	Ethyl acetate	Aqueous
Steroids	+	+	+	+	+
Alkaloids	+	+	-	+	-
Tannins	+	+	-	+	+
Phlobatannins	+	+	+	+	+
Monoterpenes	+	+	+	+	+
Flavonoids	+	+	+	+	+
Coumarins	+	+	-	-	+
Cardiac Glycosides	+	+	+	+	+
Saponins	+	+	+	+	+
Diterpenes	+	-	-	-	+
Anthraquinones	+	+	-	-	+
Phenols	+	+	+	+	+

(+) = present (−) = absent

### Total phenolic contents

Total phenolic contents in the aqueous and organic extracts of *C. angustifolia* were evaluated by plotting a standard curve using different concentrations of GAE (Gallic acid equivalent) with their respective absorbance at 700 nm. The linear regression equation [y =0.504× (R2 = 0.991)] for the calibration curve was used to evaluate the total amount of phenolic contents present in each extract which was expressed as mg of GAE/g (Table 6). The analysis showed that a considerable amount of phenolic contents were present in organic and aqueous extracts of *C. angustifolia* ranging from 0.535 ± 0.002 to 2.328 ± 0.003 mg of GAE/g of extract. The methanol and ethanol extracts have the most phenolic contents followed in order by the ethyl acetate, acetone and aqueous extracts.

**Table 6:** Quantification of phenols and flavonoids in different solvent extracts of C. *angustifolia*

S.No	Plant extracts/ chemicals (all 400 μg/mL)	Total phenolic contents (^a^mg of GAE/g of extract)	Total flavonoid content (^b^mg QE/g dried extract)
1	Methanol	2.328 ± 0.003**	5.00 ± 0.04**
2	Ethanol	1.769 ± 0.001**	3.73 ± 0.08**
3	Acetone	1.535 ± 0.004**	3.31 ± 0.06**
4	Ethyl acetate	1.318 ± 0.002*	3.70 ± 0.04**
5	Aqueous	0.535 ± 0.002**	1.29 ± 0.03**

Values are expressed as mean ± standard deviation mg of the extracts (observations of three replicates of each sample extract)

(*), and (**), significant at level *P* ≤ 0.01 and 0.001, respectively

(^a^) Gallic Acid Equivalent per gram of dry weight (mg GAE/gm). (^b^) Quercetin Equivalent per gram of dry weight (mg QE/gm)

### Total flavonoid contents

The total flavonoid contents were determined by plotting a standard curve using different concentrations of QE (Quercetin equivalent) with their absorbance at 510 nm. The linear regression equation [y =0.040× (R2 = 0.975)] of the calibration curve was used to evaluate the total amount of flavonoid contents present in each extract which is expressed as mg of quercetin equivalents per gram QE/gm (Table 6). Methanol extracts of *C. angustifolia* showed higher content of flavonoids than the other solvent extracts.

### HPLC analysis

Bioactivity-guided screening of *C. angustifolia* methanol, ethanol and ethyl acetate extracts by HPLC-MS revealed the presence of three bioactive compounds: quercimeritrin (**1**), scutellarein (**2**), and rutin (**3**).

#### Quercimeritrin (1)

C_21_H_20_O_12_, yellow amorphous powder; ^1^H NMR (400 MHz, CD_3_OD) δ: 3.48-3.98 (6H, m, H-2”, H-3”, H-4”, Ha-5”, Hb-5”), 5.25 (1H, d, J = 8 Hz, H-1”), 6.25 (1H, d, J = 2 Hz, H-8), 6.48 (1H, d, J = 2 Hz, H-6), 6.86 (1H, d, J = 8.5 Hz, H-5’), 7.63 (1H, dd, J = 8.5, 2.5 Hz, H-6’), 7.67 (1H, d, J = 2.0 Hz, H-2’). ESIMS m/z 464.38 [M]^+^, 465.38 [M + H]^+^, 487.38 [M + Na] ^+^, 463.37 [M-H]^−^.

#### Scutellarein (2)

C_15_H_10_O_6,_ reddish-brown crystals; ^1^H NMR (400 MHz, CD_3_OD) δ: 6.22 (1H, s, H-3), 6.76 (2H, d, J = 8, H-3’,5’), 7.09 (2H, d, J = 8, H-2’,6’), 7.11 (1H, s, H-8). ESIMS m/z 286.15 [M]^+^, 287.15 [M + H]^+^, 309.15 [M + Na] ^+^, 285.14 [M-H]^−^.

#### Rutin (3)

C_27_H_30_O_16_, yellowish-brown crytals; ^1^H NMR (400 MHz, CD_3_OD) δ: 1.15 (3H, d, J = 6, H-6”’), 3.27 (1H, m, H-4”’), 3.45 (1H, m, H-5”’), 3.56 (1H, dd, J = 9.5/3.5 Hz, H-3’”), 3.65 (1H, dd, J = 3.5/1.5, H-2’”), 3.26-3.53 (6H, m, H-2”, H-3”, H-4”, Ha-5”, Hb-5”), 5.13 (1H, d, J = 7.8 Hz, H-1”), 6.24 (1H, d, J = 1.8 Hz, H-6), 6.43 (1H, d, J = 2.2 Hz, H-8), 6.90 (1H, d, J = 8.0 Hz, H-3’), 7.65 (1H, dd, J = 8.0/1.8, H-2’), 7.69 (1H, d, J = 1.8 Hz, H-6’). ESIMS m/z 610.16 [M]^+^, 611.16 [M + H]^+^, 633.15 [M + Na] ^+^, 609.15 [M-H]^−^.

## Discussion

Medicinal plants are important sources of biologically active natural products which due to their curative properties have been studied for many years [25, 26]. The present study was designed to find the bactericidal potential of extracts of *C. angustifolia. A. junii*, *S. mercescens*, *E. cloacae*, *P. aeroginosa*, and *S. typhi* have been implicated in the pathogenesis of various infectious diseases [27]. Among the extracts, the methanol extract of *C. angustifolia* displayed the highest activity and a broad spectrum of activity against pathogenic bacterial strains. It was reported that the bactericidal activity was due to the presence of flavonoids found in the methanol extract [28]. It was studied that methanol and ethanol extracts of *C. angustifolia* possess significant antibacterial activity against *E. coli*, *Klebsiella pneumoniae* and *Shigella shinga* [17]. The current findings suggest that methanol, ethanol and ethyl acetate extracts are rich in flavonoids which are responsible for antimicrobial activities. Flavonoids including rutin (**3**) have been reported to have antimicrobial activities against resistant bacterial strains [29]. The aqueous extract of *C. angustifolia* was not active at the highest concentrations tested against *A. junni*, *E. cloacae* and *P. aeroginosa.* It was reported that this was due to the lower extraction of antimicrobial compounds into the aqueous extract or to minimum availability of the aqueous extract to the microorganism [30]. It was also reported that the n-butanol extract of *C. angustifolia* showed maximum antibacterial potential against *S. aureus* and *typhi* with 13 and 15 mm zones of inhibition, respectively [31]. The current study revealed that *A.junii*, *S. mercescens* and *P. aeroginosa* show resistance against the standard antibiotics amikacin and cefepime, while these bacterial strains showed sensitivity to *C. angustifolia* extracts. The current study also showed that *C. angustifolia* exhibits potential fungicidal property against *C. albicans*. It was shown earlier that saponins, active ingredients of *C. angustifolia,* were involved in antifungal activity against *Colletotridium dematium* [32]. It was reported earlier that a butanol extract of *C. angustifolia* exhibited antifungal activity against *Aspergillus terrus*, *A. flavus* and *A. niger* [31]. The antimicrobial activities of medicinal plants were due to the presence phytochemicals including saponins, terpenoids, flavonoids, phenolics, and alkaloids [33].

In the present study, antioxidant activities of *C. angustifolia* extracts revealed that methanol, ethanol, ethyl acetate and acetone exhibited significantly higher scavenging percentages and are correlated by phenolic compounds. Phenolics and flavonoids are secondary metabolites derived from tyrosine and phenylalanine with potent antibacterial and antioxidant activities [34]. It was reported that isolated flavonoids quercimeritrin (**1**), scutellarein (**2**), and rutin (**3**) have significant antioxidant activities against oxidative stress [35–37]. Note that all three have 1,2-dihydroxybenzene groups which are readily oxidized to orthoquinones, making them strong antioxidants. The aqueous extract showed a poor DPPH-scavenging activity of 67.7 %. This is because the flavonoids and phenols responsible for antioxidant activity are poorly extracted into the aqueous extract [38]. No detailed anticancer study of *C. angustifolia* has been reported earlier. This study was carried out to evaluate the anticancer potential of *C. angustifolia* and it revealed that methanol and ethanol extracts of *C. angustifolia* exhibit anticancer properties. It was reported earlier that secondary metabolites like flavonoids can be responsible for anticancer activities [29]. The current study supports the idea that the anticancer activity is due to the presence of isolated flavonoids, which were found in methanol and ethanol extracts of *C. angustifolia*. It was investigated earlier that scutellarien (**2**), extracted from *Scutellaria lateriflora,* possesses anticancer activity by significantly suppressed the proliferation of HT1080 human fibrosarcoma cells through induction of apoptosis [39]. In a similar study, it was revealed from the in vivo experiment that that the size and weight of the tumor was reduced after treatment with scutellarein (**2**) [40]. On the other hand, rutin (**3**) has been reported as anticancer agent by inducing apoptosis and cell cycle arrest in murine leukemia WEHI-3 cells [41]. It was also reported that rutin (**3**) had the potential to kill the breast cancer cells in MDA-MB-231 cell line [42]. Based on the findings, the current study supports the hypothesis that antimicrobial, anticancer and antioxidant activities of *C. angustifolia* are due to at least in part to the presence of isolated flavonoids.

## Conclusions

The present study was carried out to explore antimicrobial, antioxidant, and anticancer potential of *C. angustifolia*. Maximum antioxidant and anticancer activities were observed for methanol, ethanol and ethyl acetate extract which was correlated with phenolic and flavonoid contents. Bioactivity-guided screening of methanol, ethanol and ethyl acetate extracts revealed the presence of quercimeritrin (**1**), scutellarein (**2**), and rutin (**3**), all known to have useful bioactivities including antimicrobial, antioxidant and anticancer activities. These findings shows the importance of screening medicinal plants for antimicrobial, anticancer and antioxidant agents against resistant bacterial strains, various cancers and degenerative diseases caused by oxidative stress.

## Abbreviations

BHA: Butylated hydroxyanisole
BHT: Butylated hydroxytoluene
DPPH: 1,1-diphenyl-2-picrylhydrazyl
ESI-MS: Electronspray ionisation mass spectrometry
FBS: Fetal bovine serum
GAE: Gallic acid equivalent
HCEC: Human corneal epithelial cells
HeLa: Human cervical cancer cell line
Hep2: Human epidermoid larynx carcinoma cell line
HPLC-MS: High pressure liquid chromatogropy mass spectrometry
MCF-7: Michigan cancer foundation-7
MTT: 3-(4,5-dimethylthiazolyl)-2,5-diphenyltetrazolium- bromide
NMR: Nucleic magnetic resonance
PG: Propyl gallate
QE: Quercetin equivalent
rpm: Revolution per minute
RPMI-1640: Roswell park memorial institute-1640
TBH: t-butylhydroxytoluene
